# Heat-Induced Gelation of Legume Protein–Starch Systems: Mechanisms, Structure–Function Relationships and Food Application

**DOI:** 10.3390/gels12070562

**Published:** 2026-06-24

**Authors:** Niorie Moniharapon, Nova Geovano Setyawan Hunitetu, Lavaraj Devkota, Sushil Dhital

**Affiliations:** 1 Faculty of Science, Monash University, Clayton, VIC 3800, Australia; kalmianio@gmail.com; 2 Department of Fisheries, Faculty of Agriculture, Universitas Gadjah Mada, Yogyakarta 55281, Indonesia; novageovanosetyawanhunitetu@mail.ugm.ac.id; 3 Deakin Centre for Advanced Food Sciences, School of Exercise and Nutrition Sciences, Deakin University, Burwood, VIC 3125, Australia; lavaraj.devkota@deakin.edu.au; 4 Bioresource Processing Research Institute of Australia (BioPRIA), Department of Chemical and Biological Engineering, Monash University, Clayton, VIC 3800, Australia

**Keywords:** legume proteins, legumin/vicilin ratio, pulse starch, heat-induced gelation, meat analogues, dysphagia foods, plant-based foods

## Abstract

Plant-based food systems increasingly rely on heat-induced gelation of protein–starch mixtures, yet no focused synthesis has linked legume protein composition to mixed gel structure and function. This review critically analyses heat-induced gelation mechanisms in legume protein–starch systems, using the legumin-to-vicilin (L:V) ratio and starch origin as integrating design parameters. Legume storage proteins range from legumin-rich faba bean and *Lupinus angustifolius*, which form dense, disulfide-stabilised networks with high storage moduli, to vicilin-dominated mung bean, which produces weaker gels reliant on starch reinforcement. Pulse starches, characterised by high amylose content (24–45%), C-type crystallinity, and rapid amylose retrogradation upon cooling, act as a parallel gel-forming phase whose contribution scales inversely with protein network strength. Four protein–starch interaction modes, namely segregative phase separation, water competition, granule filler effects, and molecular complexation, jointly determine microstructure and rheological behaviour. A three-axis compositional framework defined by the L:V ratio, starch amylose content, and protein-to-starch ratio maps the gel design space. Variables favouring plant-based meat analogue performance, including high elastic modulus, yield stress, and hardness, are systematically opposed by dysphagia food requirements, including low yield stress, adequate lubrication, and soft fracture. This demonstrates that both application domains traverse the same compositional space in opposite directions. Critical research gaps include chickpea and lentil performance in meat analogue systems, mechanistic modelling of protein-matrix-mediated starch digestibility, and retrogradation kinetics during food storage.

## 1. Introduction

Plant-based foods increasingly rely on tailored gel structures to deliver desirable textures, stability and nutritional profiles, with legume ingredients playing a central role due to their high protein content, intrinsic starch fraction, and favourable environmental footprint compared with animal-derived gelling agents [1,2]. Legume proteins and starches are widely incorporated into meat and dairy alternatives, spoonable gels and dysphagia-oriented foods, where heat-induced gelation is often the dominant structure-forming process [3,4]. In such systems, thermal treatment simultaneously drives protein unfolding and network formation alongside starch gelatinisation and retrogradation, generating complex mixed gels whose structure and properties cannot be predicted from the individual components alone [5,6,7,8,9].

Several recent reviews have addressed adjacent aspects of this problem, but none focuses specifically on heat-induced legume protein–starch gels. Broad reviews on legume composition have described the physicochemical and thermal properties of legume protein, starch and dietary fibre, but treat them primarily as separate ingredients rather than as interacting components in a mixed gel network [10]. Recent reviews on plant protein/carbohydrate composite gels for plant-based meat alternatives provide a closer view, yet the protein source is treated generically rather than legume-specifically, the carbohydrate component spans hydrocolloids, fibres and starches collectively, and the application scope is limited to meat analogues [11]. Other reviews on plant protein gelation cover heat, acid, salt and pressure routes broadly, with polysaccharide co-ingredients treated as one of many influencing factors rather than as a structurally co-active phase [12]. Reviews on food gels based on polysaccharides and proteins emphasise bioactive delivery, treating starch as one of several gelling polysaccharides rather than a phase that gelatinises and retrogrades alongside the protein network [13]. Broader overviews of plant-originated gels and plant-based emulsion gels highlight general structure–function principles but remain generic with respect to legume sources and protein–starch combinations [14,15]. Across these reviews, the specific case of heat-induced legume protein–starch mixed gelation, where the legumin/vicilin balance and the starch source jointly determine network architecture, has not been systematically synthesised.

In parallel, a growing body of experimental work has begun to probe the heat-induced gelation and structural characteristics of legume protein–starch systems and is now sufficient to support a focused synthesis. Studies on chickpea and faba bean flour gels, faba bean starch–protein blends, and high-temperature gels formed from yellow pea, faba bean, mung bean and lupin protein–starch matrices report detailed rheological, microstructural and sometimes tribological data, demonstrating that protein-to-starch ratio, legume species, starch type and processing conditions can dramatically alter gel structure and functional properties [5,6,16,17]. Comparative work across pulse proteins further shows that legume-specific composition and processing history strongly govern network formation and mechanical strength [18,19,20,21]. A particularly important emerging theme is that the legumin-to-vicilin (11S/7S) ratio of the storage protein fraction governs aggregation pathways and gel-strengthening behaviour during cooling, and that this protein-side variable interacts strongly with starch amylose content and granule integrity to determine final gel properties [22]. A second emerging theme is the distinction between systems built from legume flours, where protein and starch are co-present and partially associated from the seed, versus reconstituted systems combining legume protein isolates with added starch, which can behave differently even at a matched gross composition. These threads remain scattered across formulations, processing regimes and target applications, and there is currently no focused synthesis that links molecular interactions and gelation mechanisms to microstructure, rheology, texture and nutritional behaviour in legume protein–starch gels.

The aim of this review is therefore to critically analyse heat-induced gelation mechanisms and functional properties of legume protein–starch systems in food applications, using protein subunit composition (legumin/vicilin balance) and starch origin (endogenous versus added) as integrating threads. Specifically, this review (i) summarises the physicochemical properties of legume proteins and starches that are most relevant for mixed-gel formation; (ii) describes how protein unfolding, starch gelatinisation and protein–starch interactions govern gelation pathways under thermal processing; (iii) relates the resulting gel microstructures and rheological behaviours to macroscopic texture, water-holding capacity and starch digestibility; and (iv) discusses current and potential applications of legume protein–starch gels, with a focus on plant-based meat analogues and dysphagia foods. Finally, key research gaps are identified, and future directions are proposed for the rational design of legume protein–starch gels that integrate structural, technological and nutritional targets.

## 2. Composition and Physicochemical Properties Relevant to Mixed-Gel Formation

The behaviour of a legume protein–starch gel under heat is shaped, prior to any molecular event, by the composition and physical state of the two biopolymers entering the system [7,23]. Storage protein composition and starch granule architecture are highly species-dependent and tend to dictate how protein and starch compete for water, unfold, swell, aggregate, and reassociate during thermal processing [10,16,24].

### 2.1. Legume Storage Protein: The Legumin–Vicilin Axis

Legume seed proteins are dominated by globulins, which typically account for 55–80% of total seed protein, with smaller contributions from albumins, prolamins, and glutelins [10,24,25]. Globulins are commonly classified according to sedimentation behaviour, with 7S or 8S globulins corresponding to vicilin-type proteins and 11S globulins corresponding to legumin-type proteins [8,22,26]. In pea, the 7S and 11S globulins are referred to as vicilin and legumin, respectively; therefore, structurally related proteins in other grain legumes are often described as vicilin-like and legumin-like globulins [27]. Because these protein families differ in quaternary structure, disulfide bond content, and thermal stability, the legumin-to-vicilin ratio (L:V ratio) provides a useful compositional axis for understanding heat-induced gelation.

Legumin is a hexameric protein (approximately 320–410 kDa), in which each subunit comprises an acidic alpha-chain (approximately 38–40 kDa) and a basic beta-chain (approximately 19–22 kDa) linked by a single interchain disulfide bond [8,22,26,28]; this disulfide-bridged architecture tends to confer slow heat-induced unfolding and high denaturation temperatures (*T*_d_ typically 92–100 °C in faba bean and chickpea, decreasing to approximately 85–90 °C for pea legumin under similar ionic conditions) [28,29,30,31]. In comparison, vicilin is a smaller trimeric protein (approximately 150–200 kDa) lacking cysteine residues and, therefore, intramolecular disulfide bonds, with *T*_d_ in the range of 75–87 °C across most pulses [8,17,22,32]. A third storage protein, convicilin, has been characterised in pea and faba bean but is absent from chickpea and lentil [26,28,32].

The relative abundance of legumin-like and vicilin-like proteins varies widely across legume species and cultivars [33]. These compositional differences can influence heat-induced gelation by altering protein denaturation, aggregation behaviour, and network formation [17,19,22]. As summarised in Table 1, legume species differ markedly in their dominant storage protein fractions, reported L:V ratios, thermal stability, disulfide potential, and gelation tendencies. Faba bean is generally legumin-rich and forms strong, cohesive heat-induced gels, while mung bean is dominated by 8S vicilin-type globulins and tends to form weaker gels [16,30,34,35]. Pea contains both legumin and vicilin, with reported L:V ratios varying depending on cultivar, which may partly explain its variable gelation behaviour [22,28,36]. Lupin provides another useful contrast because its major globulins are described as conglutins rather than vicilin and legumin. For example, *Lupinus angustifolius*, which is relatively richer in α-conglutin, has been associated with firmer heat-induced gels than *Lupinus albus*, which contains a higher proportion of β-conglutin [17,37].

Overall, legumin-rich systems tend to form dense, disulfide-stabilised networks with high gel strength upon cooling, whereas vicilin-rich systems aggregate predominantly through hydrophobic interactions, producing weaker but more rapidly setting gels [16,17,19,22,29,34]. However, the L:V ratio should be treated as a compositional guide rather than the sole determinant of gelation, because minor seed components and processing history can also affect protein hydration, unfolding, aggregation, and water distribution during heating [22,36,37,38].

**Table 1:** Comparative properties of major legume storage proteins relevant to heat-induced mixed-gel formation.

Legume	Globulin (% Total Protein)	Dominant Fraction	Reported Globulin Ratio	Denaturation Temperature *T*_d_ (°C, DSC)	Disulfide Content	Gelation Tendency
*Pea (Pisum sativum)*	~70–75 [22,39]	Legumin + vicilin (convicilin) [22,36]	V:L ≈ 0.5–1.7, (cultivar-dependent) [26]	~69–77 [22,39]	Low–moderate (legumin only; vicilin lacks Cys) [22,39]	Variable; concentration- and cooling-rate dependent [22,39]
*Faba bean (Vicia faba)*	~70–80 [40,41]	Legumin-rich [40,42]	L:V ≈ 1:1 to 3:1 across cultivars [40,42,43]	76.5–83.8 (vicilin), 85–95.3 (legumin); ionic-strength dependent) [34]	High (legumin) [29,44]	Strong, cohesive, heat-stable [16,29]
*Chickpea (Cicer arietinum)*	~50–60 [45,46]	Mixed [47]	V:L ≈ 0.63–2.23 [47]	~80.5 (vicilin) ~90.8 (legumin) [48]	Low–moderate [49]	Strong, network forming and condition-dependent [50]
*Lentil (Lens culinaris)*	~70 [31]	Mixed, vicilin-leaning [51]	V:L ≈ 2.21 to 2.38 [52]	~84.9 [53]	Low/minor [19]	Moderate, cooling-set [19]
*Mung bean (Vigna radiata)*	~60–85 [54,55]	Vicilin-dominant (8S) [56]	V:L ≈ 11.7:1 [57,58]	80.8–83.0 [59]	Low, 8S lacks disulfide bonds [57]	Weak, prone to phase separation [16,60]
*Lupin (Lupinus albus)*	90 [61]	β-conglutin (vicilin-like) [17,37]	α:β = 1:2, equivalent to L:V ≈ 0.5 [17]	88 (β-conglutin), 101 (α-conglutin) [17,37]	Low, β-rich, mainly hydrophobic/H-bonded [17]	Softer, quick-set; higher β-conglutin gives elevated WHC [17]
*Lupin (Lupinus angustifolius)*	90 [61]	α-conglutin (legumin-like) [17,37]	α:β = 2:1, equivalent to L:V ≈ 2.0 [17]	88 (β-conglutin), 101 (α-conglutin) [17,37]	Higher; α-rich, disulfide-linked subunits [17]	Strong, firm, heat-stable [17]

L:V, legumin-to-vicilin ratio; V:L, vicilin-to-legumin ratio; α:β, α-conglutin-to-β-conglutin ratio; DSC, differential scanning calorimetry; *T*_d_, denaturation temperature; WHC, water-holding capacity. Values are literature ranges and may vary with cultivar, protein fraction, extraction method and processing conditions.

### 2.2. Legume Starches: High-Amylose, C-Type Granules

Alongside the storage protein fraction, starch granule architecture provides a second compositional axis controlling heat-induced mixed-gel formation in legume-based systems. In starch-granule-rich grain legumes, starch forms a substantial seed fraction. The pulse starch literature reports that starch accounts for approximately 22–45% of seed dry matter, while more recent data for pea, lentil, and faba bean report approximately 40.8–51.2% of dry seed mass [62,63]. This makes starch an unavoidable co-occurring biopolymer in flour-based legume gels and an important component in formulated legume protein–starch systems [63]. Legume starches differ from many cereal and tuber starches in amylose content, crystalline polymorphism, gelatinisation behaviour, pasting viscosity, and retrogradation tendency [62,63,64]. These properties are summarised across major legumes in Table 2.

The main structural feature of many legume starches is their relatively high amylose content. Common pulse starches, including pea, lentil, faba bean, and chickpea starches, are generally amylose-rich, although reported values vary with species, cultivar, isolation method, and analytical approach [63]. The recent review literature also highlights faba bean and mung bean starches as amylose-rich legume starches, with faba bean starch commonly associated with C-type crystallinity and amylose contents around 32–40% [72], and mung bean starch reported to contain a broad amylose range of approximately 16–45% [66]. Amylose is the most linear starch fraction, whereas amylopectin is highly branched [73]. This distinction is important because linear amylose chains can leach from swollen granules during heating and reassociate into ordered double-helical and partially crystalline structures during cooling. These reassociation events contribute to retrogradation, setback viscosity, final viscosity, and gel firmness [62,63,66,67]. 

Another important feature of legume starches is their C-type crystallinity. Crystallinity type describes the packing arrangement of starch chains inside the granule and is commonly determined by X-ray diffraction or wide-angle X-ray diffraction [62,63]. Starches are commonly classified into A-, B-, and C-type polymorphs, with cereal starches generally showing A-type crystallinity, tuber starches commonly showing B-type crystallinity, and legume starches showing a mixed C-type pattern [74]. C-type crystallinity combines features of both A- and B-type polymorphs and has been reported across major legume starches, including pea, lentil, faba bean, chickpea, mung bean, black gram, and pigeon pea [62,63,65,75]. In a comparative study of six legume starches, C-type diffraction patterns were observed with relative crystallinity values of approximately 27.2–33.5%, while broader pulse starch reviews report a wider range of approximately 19–36% depending on species, cultivar, moisture condition, and analytical method. This crystalline organisation influences water penetration, granule swelling, and the extent of structural disruption during heating [62,63,65]. 

Legume starches also tend to retrograde rapidly and extensively upon cooling. Short-term retrogradation is mainly driven by amylose reassociation, whereas longer-term storage may involve amylopectin recrystallisation [62,63,76]. However, starch behaviour is not determined by amylose content alone. Mung bean starch is a useful example because it can generate exceptionally high peak viscosity, often exceeding 6000 mPa.s, but also shows high breakdown and strong retrogradation behaviour that can produce firm yet brittle gel networks [62,66,67,68,77]. Chickpea starch provides a different case because its amylose content and lipid fraction can vary with cultivar and extraction conditions, potentially influencing swelling, gelatinisation, pasting, retrogradation, and digestibility [78]. In starch systems containing sufficient amylose and lipids, amylose lipid inclusion complexes can form during heating, producing V-type crystalline structures and resistant starch type V fractions that are relevant to starch digestibility and glycaemic design [76,79,80]. Overall, amylose content, crystallinity type, granule swelling behaviour, and retrogradation tendency define the starch-side structural properties that must be considered.

## 3. Mechanisms of Heat-Induced Gelation in Legume Protein–Starch Systems

**Figure 1:**
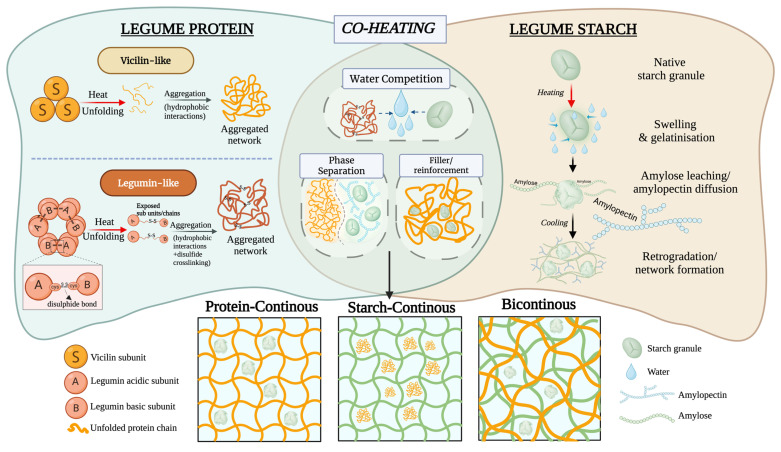
Mechanistic overview of heat-induced gelation in legume protein–starch systems, based on concepts reported in the literature [81,82]. In the network schematics, the colour coding distinguishes protein-associated structures from starch-associated structures, as indicated in the legend. (Created in BioRender. Moniharapon, N. (2026). https://BioRender.com/qcgoafp).

The mixed gel that emerges from a heated legume protein–starch system is the integrated outcome of three concurrent processes: protein unfolding and aggregation, starch granule swelling and gelatinisation, and the interactions between protein and starch as both compete for water and physical space [7,9,33]. These coupled thermal and interaction pathways are summarised schematically in Figure 1.

### 3.1. Protein-Side Events on Heating

Heat-induced gelation begins when heating destabilises the folded structure of globular legume storage proteins. As temperature increases, the proteins denature and buried hydrophobic regions become exposed to the surrounding aqueous phase, creating reactive surfaces that promote aggregation [83]. These newly exposed sites initiate heat-driven protein–protein association, first forming smaller aggregates and then larger clusters that connect into a continuous protein network [10,84]. Thus, protein-side heat-induced gelation involves a sequence of thermal unfolding, association, aggregate growth and network formation rather than denaturation alone [84].

The dominant aggregation pathway depends on the reactive groups exposed during heating. Legumin-type proteins possess acidic and basic subunits that are structurally connected by disulfide bonds [85]. However, pea legumin specifically has been reported to form heat-induced gels mainly through hydrophobic interactions and hydrogen bonding, although disulfide bonds may contribute under some cooling or network-strengthening conditions [8]. In contrast, vicilin-type proteins contain fewer cysteine residues available for disulfide formation, and their heat-induced aggregation similarly relies strongly on non-covalent interactions such as hydrophobic association and hydrogen bonding [28,85]. These differences in protein composition and reactive group exposure contribute to variation in final gel properties, including gel stiffness and resistance to deformation, across legume protein systems [86]. 

These molecular pathways describe the idealised sequence for heat-induced protein gelation, but commercial legume protein ingredients may not begin from a fully native state. Industrial processes, including isoelectric precipitation and spray drying, can substantially denature proteins prior to laboratory gelation tests [83]. As a result, heating may reinforce or rearrange an already aggregated protein system rather than initiate a classical sol-to-gel transition. The commercial extraction method has also been shown to play a major role in determining the gelation pathway taken [18]. This network formation or reinforcement can be tracked rheologically through the storage modulus (G′), which reflects elastic or solid-like behaviour, and the loss modulus (G″), which reflects viscous or liquid-like behaviour; higher G′ values indicate a stronger intermolecular network [18,78]. In mixed protein–starch systems, the timing of protein network formation becomes important because starch also undergoes heat-induced structural changes, as discussed in the next section.

### 3.2. Starch-Side Events on Heating

As outlined in Section 2.2, legume starches are generally characterised by relatively high amylose contents and C-type crystalline organisation, but their contribution to heat-induced mixed gelation depends on how these structural features respond during thermal processing [62,87,88,89,90]. During heating in excess water, legume starch granules hydrate and swell as heat and moisture progressively disrupt the ordered organisation of the semi-crystalline granule structure [87,88,91,92,93]. In legume starches, this transition can occur through staged structural changes, beginning with water absorption and swelling at lower temperatures, followed by structural rupture at intermediate temperatures and melting or reorganisation at higher temperatures [91]. At the lamellar level, the amorphous region swells first, followed by hydration and melting of the crystalline region, indicating that gelatinisation is a progressive structural transition rather than a single temperature event [62,91].

As gelatinisation proceeds, the semi-crystalline structure becomes increasingly disordered, granule swelling increases, and amylose leaches from the swollen granule into the surrounding starch water phase [87,88]. This transition changes starch from a compact granular particle into a hydrated, deformable and viscosity-building phase because both granule expansion and dissolved starch molecules contribute to increased paste viscosity [87,88]. Therefore, the starch-side mechanism is not only defined by gelatinisation temperature, but also by the degree of granule swelling, the extent of amylose leaching, and the amount of granular structure that remains after heating [87,88,91]. 

The distinctive behaviour of legume starches lies less in a fundamentally different gelatinisation pathway and more in the balance between C-type crystalline organisation, relatively high amylose content, controlled swelling and residual granule integrity during heating [94]. Most legume starches are C-type starches, containing both A-type and B-type crystalline features, and this mixed crystalline organisation contributes to their distinctive swelling, gelatinisation, retrogradation and pasting behaviour. Pulse starches also generally contain higher amylose levels than many cereal and tuber starches, which can restrict excessive swelling during heating while supporting stronger molecular reassociation during cooling [78,88,95]. However, legume starches should not be treated as a uniform group, because the botanical source, amylose content, crystalline organisation, granule integrity and heating conditions can produce different swelling and pasting responses. Thus, pulse starch behaviour should be interpreted through the combined effects of amylose content, C-type crystallinity, granule swelling, amylose leaching and paste stability rather than gelatinisation temperature alone.

During cooling, leached amylose reassociates through starch chain interactions and recrystallisation, initiating retrogradation and contributing to setback behaviour [64,88]. In pulse starches, retrogradation is influenced by the amount of amylose leached during gelatinisation, amylose–amylose interactions, amylose–amylopectin interactions and the mobility of starch chains during storage [64]. This reassociation process increases crystallinity, gel firmness and network formation in the cooled starch phase [64,96]. Therefore, legume starches can contribute to final gel firmness even when swelling during heating is relatively controlled, because the cooling stage allows leached starch chains to reinforce the matrix through retrogradation. Thus, the starch-side contribution to heat-induced gelation involves gelatinisation and amylose leaching during heating, followed by molecular reassociation and network reinforcement during cooling [64,88,91,92].

### 3.3. Protein–Starch Interactions During Co-Heating

As described in Section 3.1 and Section 3.2, heating promotes legume protein unfolding and aggregation while also driving starch hydration, swelling, gelatinisation, amylose leaching and subsequent retrogradation. During co-heating, these pathways occur in the same aqueous environment and therefore influence each other rather than proceeding independently. This is particularly important because the thermal transitions of legume proteins and starches often overlap. Legume storage proteins commonly denature across approximately 70–100 °C depending on species, protein composition and ionic conditions, while legume starches, which are commonly characterised by C-type crystallinity and relatively high amylose contents, gelatinise over a broad range that may begin around 60 °C and extend beyond 80 °C in intact seed or flour systems [6,16,97,98]. Thus, protein aggregation and starch swelling may occur concurrently, causing both phases to compete for water and physical space. The resulting mixed gel is shaped by four overlapping mechanisms: phase separation, water competition, starch granule filler effects and possible molecular complexation. 

First, phase behaviour determines how protein and starch are spatially arranged during heating. At near-neutral pH, many legume globulins carry a net negative charge above their isoelectric points, whereas starch granules are largely neutral. This makes strong electrostatic complexation less likely and can favour segregative phase separation, where protein-rich and starch-rich domains form within the same gel matrix [99,100]. In protein-rich systems, the gel may become protein-continuous, meaning that the protein network forms the main connected phase while swollen starch granules are dispersed within it. In starch-rich systems, the opposite arrangement may occur, producing a starch-continuous structure in which swollen or gelatinised starch forms the main connected phase and protein aggregates occupy the spaces between granules [99,100]. At intermediate compositions, both phases may become partially continuous, producing a bicontinuous or interpenetrating network in which both protein and starch contribute to the final structure [2,3,9]. Such composition-dependent transitions have been observed in mung bean protein–starch hydrogels and faba bean protein–pea starch gels [97,101].

Second, protein and starch compete for water during heating. Starch granules require water to hydrate, swell and gelatinise, while proteins also require water to unfold, move and aggregate into a network. As starch granules absorb water, they can draw water away from the surrounding protein phase, locally concentrating proteins and promoting aggregation [80,102]. Similar redistribution has been quantified in beans and chickpeas during cooking, where water was mainly associated with proteins at the beginning of heating but became increasingly associated with starch as cooking progressed [95]. Conversely, if protein aggregation occurs before extensive starch hydration, the developing protein network may surround starch granules, restrict water access, reduce swelling and limit gelatinisation [16,97,101,102]. Therefore, the relative timing between protein aggregation and starch gelatinisation influences whether the final gel is protein-dominated, starch-dominated or mixed. 

Third, intact or partially swollen starch granules can act as fillers within the protein network. When granule–matrix adhesion is strong, starch granules may behave as active fillers that reinforce the network and increase small-deformation stiffness, commonly reflected by a higher storage modulus, G′ [6,97]. However, when adhesion is weak, starch granules may behave as inactive fillers that interrupt network continuity and reduce large-deformation strength [6,97]. Therefore, starch addition does not automatically strengthen a mixed gel. Its effect depends on granule swelling capacity, amylose content, residual granule integrity, surface compatibility and the degree of phase compatibility with the protein matrix [6,97,103]. In a model pea protein–starch system containing starches with different amylose contents, amylose level influenced the microstructure, water partitioning and mechanical properties, with high-amylose starch maintaining greater granule integrity and favouring a more protein-dominated network after heating [103].

Fourth, molecular complexation may contribute under specific conditions, particularly when amylose leaches from gelatinised starch granules, and suitable hydrophobic or amphiphilic ligands are present [23,80,104,105]. Leached amylose can encapsulate lipids or other hydrophobic guest molecules within its helical cavity, forming V-type inclusion complexes [23,80,104,105]. In systems containing legume proteins, starch and fatty acids, V-type crystalline structures have been detected by X-ray diffraction (XRD), Fourier-transform infrared spectroscopy (FTIR) and Raman spectroscopy, and protein presence was reported to improve starch–lipid complex ordering [105]. These complexes can reduce starch digestibility by limiting enzyme access to the starch chain and may also affect retrogradation during storage [23,80,104,105]. However, molecular complexation should be interpreted as a possible secondary pathway rather than a universal mechanism in all legume protein–starch gels. Phase separation, water competition and filler effects are often more directly responsible for the observed microstructure and rheology. 

### 3.4. A Unifying Framework for Heat-Induced Legume Protein–Starch Gels

**Figure 2:**
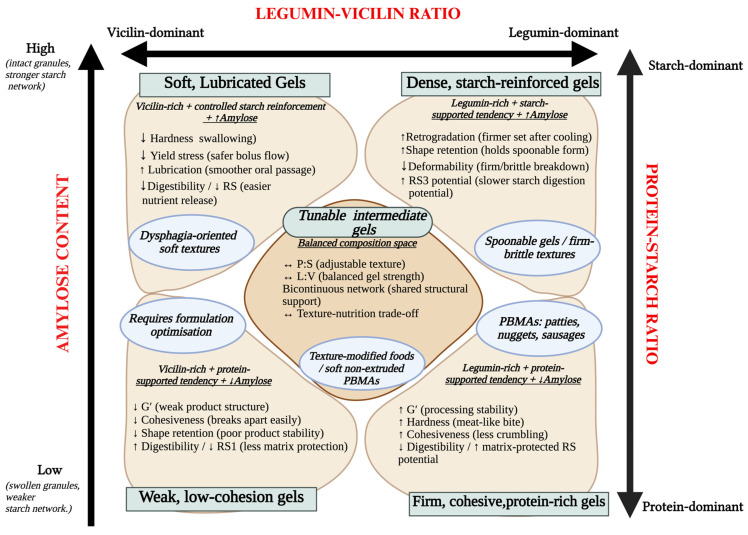
Three-axis compositional design map for heat-induced legume protein–starch gels. In the figure, ↑, ↓, and ↔ indicate increase, decrease, and adjustable/bidirectional compositional effects, respectively. Abbreviations: L:V, legumin-to-vicilin ratio; P:S, protein-to-starch ratio; G′, storage modulus; RS, resistant starch; RS3, retrograded resistant starch; PBMAs, plant-based meat analogues. (Created in BioRender. Moniharapon, N. (2026). https://BioRender.com/qcgoafp).

The gel architecture in legume protein–starch systems can be mapped onto three orthogonal compositional axes: the legumin-to-vicilin ratio, the amylose content and granule integrity of the starch, and the protein-to-starch ratio. Four limiting outcomes emerge: (1) a continuous protein matrix with embedded starch granule fillers, favoured at high protein-to-starch ratios with legumin-rich proteins and moderate-amylose starches [14,15,16]; (2) a continuous starch matrix with dispersed protein aggregates, favoured at low protein-to-starch ratios with vicilin-rich proteins and high-amylose, strongly retrograding starches; and (3) a bicontinuous network at intermediate ratios, targeted in dysphagia and structured plant-based food applications [23,106]. This framework functions as the operational map for both applications discussed in Section 5 and summarised in Figure 2.

To facilitate interpretation, Figure 2 represents the three compositional axes on a two-dimensional grid. The horizontal axis denotes the legumin-to-vicilin ratio, ranging from vicilin-dominant compositions on the left to legumin-dominant compositions on the right. The two vertical axes are plotted concurrently, with starch amylose content and granule integrity increasing in parallel with the protein-to-starch ratio from bottom to top; the lower boundary therefore corresponds to protein-dominant, low-amylose conditions, whereas the upper boundary corresponds to starch-dominant, high-amylose conditions. The four corner regions represent combinations of these compositional extremes, while the central region denotes the tunable, bicontinuous intermediate gels described above.

Although the three-axis framework defines the compositional design space, the final gel architecture also depends on the processing environment, which can shift the relative timing between protein unfolding and starch gelatinisation [97]. Changes in pH alter protein charge density and solubility, particularly near the isoelectric point, promoting aggregation and a more protein-continuous structure [5]. Ionic strength further modulates this pH effect, exerting only a minor influence on gelation at a neutral-to-alkaline pH but a substantially larger effect under acidic conditions [107]. Because protein and starch compete for the same available water pool, these pH- and ionic-strength-driven shifts can redirect hydration toward or away from swelling starch granules, reinforcing or offsetting the compositional axes described [97,102]. Mechanical shear, as encountered in high-moisture extrusion, can similarly disrupt swollen starch granules [108] and align protein domains into a more anisotropic, phase-separated structure [109]. Accordingly, pH, ionic strength, and shear history should be regarded as process-dependent modifiers of the three-axis framework rather than additional compositional axes.

## 4. Structure–Function Relationships of Legume Protein–Starch Gels

### 4.1. Microstructure

Confocal laser scanning microscopy (CLSM) with selective fluorescent staining provides direct evidence of phase distribution in hydrated gels: rhodamine B can be used to visualise the protein phase, while starch can be visualised using fluorescent labels such as 8-aminopyrene-1,3,6-trisulfonic acid (APTS) or fluorescein isothiocyanate (FITC). SEM, polarised light microscopy, SAXS, and XRD provide complementary structural information at different length scales [16,18,61,75,97,110,111]. Studies on faba bean protein with pea starch confirm the framework predictions: at high protein-to-starch ratios, the protein forms a continuous matrix with swollen starch granules dispersed as inclusions; at low ratios, the starch network becomes load-bearing [14,15,93]. Studies on lentil starch–lentil protein composite pastes and gels also demonstrate that the microstructure changes with starch and protein composition, indicating that legume protein–starch systems should be interpreted through both phase arrangement and matrix continuity rather than composition alone [112]. More recent work on faba bean protein–pea starch composite gels further shows that changing the ratio of faba bean protein to pea starch affects textural, rheological and microstructural properties, providing a direct example of how legume protein and starch fractions form different structural organisations depending on formulation ratio [97]. In commercial yellow pea, faba bean, and mung bean protein–starch systems, differences in post-heating aggregate morphology were also consistent with differences in rheological and textural behaviour, although these systems should be interpreted together with differences in protein source, starch substitution level and high-temperature processing conditions [16].

### 4.2. Rheological Behaviour

Available studies suggest that protein composition can influence rheological behaviour, although this effect is also shaped by starch substitution, protein concentration, extraction history and processing temperature. In commercial legume protein–starch systems, faba bean showed higher G′ than yellow pea and mung bean under comparable RVA heating conditions, while lupin systems also showed different final G′ values depending on the relative abundance of legumin-type and vicilin-type fractions [15,16,93]. In mixed systems, changes in storage modulus (G′) and loss modulus (G″) should be interpreted as the combined outcome of protein aggregation, starch gelatinisation, water redistribution and network rearrangement during cooling. This interpretation is supported by same-legume composite systems: lentil starch–lentil protein gels showed composition-dependent rheological and microstructural behaviour as the starch-to-protein ratio changed [112], while faba bean starch–protein mixtures showed higher pasting viscosity, higher G′, lower loss tangent (tan δ = G″/G′), and stronger gel formation as starch content increased [6]. More recent legume-derived mixed systems, including faba bean protein–pea starch and mung bean starch–mung bean protein hydrogels, further show that starch/protein ratio, starch gelatinisation state and protein aggregation state can alter phase behaviour and gel network properties [97,101].

### 4.3. Texture and Tribology

Texture profile analysis (TPA) and tribology provide complementary information on the mechanical behaviour of legume protein–starch gels because they probe different deformation modes [113,114]. TPA applies a double-compression cycle to cooled gels and reports bulk deformation parameters such as hardness, cohesiveness, springiness, gumminess and chewiness. Tribology, in contrast, measures friction between sliding surfaces separated by a thin food film and is therefore more relevant to lubrication behaviour during oral-type deformation [115,116]. These methods should therefore be interpreted together rather than treated as interchangeable measurements.

In TPA, hardness is usually defined as the peak force during the first compression cycle and reflects the combined effects of network continuity, starch granule swelling, amylose reassociation during cooling, and adhesion between protein-rich and starch-rich domains [23,117]. However, hardness should not be interpreted simply as a direct measure of a stronger protein network. Starch–protein composite studies show that protein addition can either weaken or reinforce gel structure depending on solid content, phase continuity and interfacial compatibility [118,119]. More directly, legume-based systems show similar composition-dependent behaviour. Lentil starch–lentil protein gels, faba bean starch–protein mixtures, and faba bean protein–pea starch composite gels all demonstrate that changing the starch-to-protein ratio modifies rheological, textural and microstructural properties [6,97,112]. Thus, gel hardness is better interpreted as an integrated response to phase continuity, starch reinforcement, protein aggregation and interfacial adhesion [120].

Other TPA parameters provide related information on repeated deformation. Cohesiveness reflects the ability of the internal structure to withstand a second compression, while springiness reflects height recovery after deformation [117,121]. Gumminess and chewiness are derived from hardness, cohesiveness and springiness, and therefore should be treated as integrated descriptors rather than independent structural quantities [121,122]. Because TPA values are affected by sample geometry, strain level, compression speed and preparation history, direct numerical comparison across studies should be made cautiously and supported by rheological or microstructural evidence [111,120,122].

Tribology adds an oral-relevant perspective because gel breakdown produces a hydrated bolus containing protein aggregates, swollen starch granules, starch fragments, leached amylose and released water. These components can modify friction by changing viscosity, forming boundary layers or interacting with salivary proteins and oral surfaces [113,115]. Therefore, a firm gel under TPA does not necessarily show high friction if it releases sufficient hydrated starch or water during breakdown, while a softer gel may still feel adhesive or astringent if protein-rich surfaces interact strongly with saliva [123]. Reading TPA and tribology together, alongside microstructural evidence, provides a more balanced interpretation of legume protein–starch gel structure–property relationships [124,125].

### 4.4. Water-Holding Capacity and Starch Digestibility

Water-holding capacity (WHC) in legume protein–starch gels is governed by network cross-link density, the water-binding affinity of each phase, and water partitioning between phases during heating and cooling [95,118,121,126]. Confocal laser scanning microscopy (CLSM) image analysis of maize starch–pea protein composites confirms uneven inter-phase water distribution [111] while time-domain nuclear magnetic resonance (TD-NMR) measurements on faba bean systems show that water migrates from protein to starch as granules swell and gelatinise during heating [95]. WHC tends to increase with starch substitution in faba bean systems, is more variable in mung bean systems, where high amylose content tends to enhance water binding, but rapid granule rupture can expel water during processing, and syneresis during storage is most pronounced in starch-dominated gels as amylose retrogradation continues post-processing [21,63,64,83,127,128].

A defining nutritional feature of legume-based foods is slow starch digestibility, manifested as elevated slowly digestible starch and resistant starch fractions in vitro and a lower glycaemic index in vivo [78,129,130,131]. Legume protein–starch gels can, in principle, generate three resistant starch classes simultaneously: RS-3 from retrograded amylose, RS-V from amylose–lipid complexes (most prominent in chickpea-based systems), and RS-1 from protein-matrix entrapment of partially gelatinised granules [129,130]. Evidence for protein-matrix entrapment as a digestibility barrier comes from studies on intact legume cotyledon cells [129] and formulated gel systems where increased protein content raised resistant starch content in 3D-printed corn starch–salmon protein matrices and protein adsorption onto granule surfaces tended to restrict swelling and reduce glycaemic response in soy and whey protein composites. This structural interference is, however, bidirectional. A denser or more compact gel matrix, such as that produced when starch reinforcement increases network density and reduces pore size, has been shown to impede enzyme diffusion through steric hindrance, lowering gastric-phase pepsin digestibility of the embedded protein even where overall intestinal digestibility remains largely unaffected [132,133,134]. This trade-off is particularly relevant for elderly and dysphagia-oriented formulations, where the soft, protein-continuous structures favoured for safe swallowing are also expected to maximise protein release and absorption.

## 5. Application

### 5.1. Plant-Based Meat Analogues

Plant-based meat analogues (PBMAs) represent one of the largest commercial applications of legume protein–starch systems [3,134,135,136,137]. Heat-induced gelation contributes as the primary structuring step in non-extruded products (patties, nuggets, sausages), as a post-extrusion setting step during solidification in the cooling die of high-moisture extrudates, and as a conditioning step during product reheating [135,138,139].

#### 5.1.1. Legume-Specific Performance

Different legume proteins offer different structuring roles in PBMAs because their legumin-to-vicilin balance affects gel strength, heat stability, and interaction with starch during heating and cooling. Therefore, legume selection should be linked to the target product texture, from firm and cohesive patties or nuggets to softer or spreadable meat analogue formats [17,135,137]. In these systems, starch can support texture by acting as a filler, binder, or secondary gel-forming phase depending on starch type, protein-to-starch ratio, and cooling behaviour [11,136,140].

Pea protein is the dominant commercial choice for PBMAs, valued for its balance of solubility, water-holding, and gelation behaviour, though its vicilin-dominated globulin profile tends to produce gels of lower strength than legumin-rich alternatives [9,20,36]. In model meat analogue systems, a 70:30 thermally inhibited waxy starch-to-pea protein ratio at 47% total solids most closely matched commercial meat analogue texture, suggesting that pea protein may shift from an active network-forming component to a more passive filler-like phase after refrigeration when starch dominates the final structure [140]. This indicates that pea protein is suitable for intermediate PBMA textures, but its final performance is highly dependent on starch type, solids content, and post-heating cooling behaviour.

Faba bean protein is increasingly relevant for firmer PBMA formats because its legumin-rich composition supports stronger and more thermally stable heat-induced gels than pea protein at comparable concentrations [29,34,141,142]. This makes faba bean suitable for products requiring bite resistance and shape retention, such as patties, nuggets, and sausages, although beany flavour and inadequate juiciness remain persistent sensory challenges [141,142]. In contrast, mung bean protein forms weaker pure-protein gels because it is dominated by vicilin-type globulins, but its high-amylose starch fraction can provide additional reinforcement through rapid retrogradation during cooling [143,144]. Mung bean-based systems, therefore, require processing conditions that coordinate starch gelatinisation with protein aggregation; the final structure may become weak, brittle, or poorly integrated [59,143,144]. Lupin provides a different design opportunity because *L. angustifolius* is relatively richer in α-conglutin, which is legumin-like, whereas *L. albus* contains more β-conglutin, which is vicilin-like [17,37,145,146]. This species-level contrast allows *L. angustifolius* to be positioned toward firmer PBMA formats and *L. albus* toward softer or spreadable products, although deliberate starch source selection is needed because lupin contains little endogenous starch [17,37,145,146].

Chickpea and lentil provide a final group of promising but less explored PBMA ingredients. Chickpea and lentil have received less PBMA-focused attention despite legumin-leaning compositions and moderate gel strengths [5,19,147,148]; chickpea’s higher residual lipid may generate amylose–lipid complexes that simultaneously elevate resistant starch content and contribute meat-analogue texture, a combination not yet systematically explored [78,148,149].

#### 5.1.2. Starch Function and Design Considerations in PBMAs

Starch contributes to PBMA structure as a filler, binder, and secondary gel-forming phase. As a filler, swollen starch granules can increase stiffness and water retention within the gel matrix [140,150]. As a binder, gelatinised starch increases continuous-phase viscosity and helps hold dispersed particles together, which can improve cooking stability and reduce cooking loss [11,140,151]. As a secondary gel-forming phase, leached amylose can reassociate during cooling and strengthen the final structure through retrogradation, which is particularly important when starch is expected to provide more than passive filler reinforcement [11,119,144].

However, starch addition creates important formulation trade-offs. Reduced cooking loss can indicate better water retention and structural stability, but excessive water immobilisation may limit moisture and lipid release during chewing, contributing to dryness and inadequate juiciness [140,141,150]. Similarly, amylose retrogradation can improve firmness and cohesiveness, but excessive retrogradation may produce brittle or overly hard textures that do not match desirable meat analogue bite [17,120]. Therefore, starch selection in PBMAs should be guided not only by gel strength, but also by the target balance between firmness, cohesiveness, juiciness, cooking stability, and oral moisture release.

Persistent performance gaps remain. PBMAs often struggle to reproduce the dynamic moisture and fat release of meat, even when cooking stability is improved [141,142,151]. Hardness can also become difficult to reduce without weakening cohesiveness, especially in strongly retrograding starch-containing systems [136,152,153]. In addition, off-flavours such as beany, bitter, and grassy notes may remain after extraction and thermal processing, meaning that starch-based texture optimisation still needs to be combined with flavour and sensory design [135,137,142].

### 5.2. Dysphagia Foods

Dysphagia affects elderly populations and patients with neurological conditions, stroke, or head-and-neck cancer, and texture-modified foods are commonly used to improve swallowing safety and nutritional intake [154]. Heat-induced legume protein–starch gels are relevant to this application because their structure can be adjusted through protein concentration, starch type, water distribution, and cooling behaviour to produce soft, cohesive, and lubricating textures [3,155,156,157]. Unlike PBMAs, where strong gel networks and bite resistance are often desirable, dysphagia foods require controlled softness, smooth bolus formation, and low friction during oral processing.

#### 5.2.1. Evidence for Legume Protein–Starch Dysphagia Gels

The most systematic demonstration was obtained from studies in which pea protein (0–10 wt%) and pea starch (0–7.5 wt%) concentration were varied in heat-induced gels followed by evaluation of rheology, tribology, and International Dysphagia Diet Standardisation Initiative (IDDSI) compliance [3]. Starch addition of at least 2.5 wt% promoted a transition from fluid-like to solid gel-like behaviour; all pea starch-pea protein matrices exhibited lower friction coefficients than buffer alone, with 10% protein combined with 7.5% starch achieving a friction coefficient of 0.01 or below in the boundary regime, attributed to combined interfacial boundary-layer formation and viscous lubrication via the hydrated gel network [3]; and IDDSI testing confirmed compliance with Levels 2, 3, 4, and 6, demonstrating broad applicability across dysphagia severity levels through composition variation alone [3]. A comparative study by Cai et al. 2026 using soy protein isolate with nine different starches found that only rice and cassava starch formulations achieved IDDSI Level 5, whereas the legume starches (pea and mung bean) produced gels too firm or brittle for that target [158] highlighting an important caveat: the high amylose content and rapid retrogradation that benefit meat analogue applications may result in excessive firmness for the softest dysphagia texture levels.

#### 5.2.2. Emulsion-Filled Gels, 3D Printing, and Elderly Nutrition Considerations

Emulsion-filled gels (EFGs) address the persistent challenge of inadequate caloric and protein density in texture-modified diets [154,155,159]. A recent study developing pea protein-kappa-carrageenan EFGs containing 20% oil and 10% pea protein demonstrated that all three interfacial compositions tested met IDDSI Level 6 criteria, but differed substantially in mechanical properties and in vitro digestibility, highlighting that IDDSI classification alone is insufficient to distinguish mechanically distinct formulations [156]. Exogenous lipids participate actively in this ternary protein–starch–lipid system rather than acting as an inert filler. In emulsion-filled pea protein–potato starch gels, oil droplet incorporation altered Young’s modulus, fracture stress, and adhesiveness, with the direction and magnitude of these changes depending on whether the starch formed a particulate or continuous polymer network [160]. Comparable behaviour has been reported in legume-starch-based adipose tissue mimetics, where pea starch combined with chickpea flour and 40% oil produced a starch network that encapsulated discrete oil pockets, reproducing the textural and thermal stability of animal adipose tissue [161]. These findings suggest that exogenous lipids constitute a third structural axis, alongside protein-to-starch ratio and starch type, which may further modulate water competition, phase continuity, and the lubricating boundary layer relevant to tribological performance in PBMA and EFG design.

Legume protein-based EFGs may therefore simultaneously deliver dysphagia-compliant texture, high protein density, and improved in vitro digestibility [156]. Three-dimensional printed pea protein gels with physical modifications (ultrasound, microwave, heating) have been shown to reach IDDSI Levels 4–5 with customisable shapes that may improve patient acceptance [162,163]. For elderly populations, they may approach postprandial muscle protein synthesis rates seen with dairy proteins when amino acid composition and dose are optimised, though plant proteins generally elicit lower anabolic responses than animal-derived counterparts [163]; hydrothermal processing may additionally increase protein digestibility by denaturing protease inhibitors [72,164]. Legume protein–starch gels, designed to simultaneously satisfy IDDSI compliance and sarcopenia prevention targets, therefore represent a strategically important food category that addresses two major and concurrent public health challenges [159,165].

## 6. Challenges and Limitations

Heat-induced legume protein–starch systems have great potential; however, several research gaps remain. (1) For legume-specific performance, chickpea and lentil, despite legumin-leaning compositions and moderate gel strengths, remain underexplored in meat analogue contexts; chickpea’s amylose–lipid complex formation represents an untapped lever for simultaneously engineering texture and resistant starch content. Tribological data, increasingly available for pea systems, have not been extended systematically to faba bean, mung bean, or lupin matrices. (2) Mechanistic models connecting protein- matrix architecture to starch digestibility in formulated gel systems, as opposed to intact cotyledon cells, are lacking. Long-term stability of retrograded starch networks within protein gels during refrigerated and frozen storage is poorly characterised. (3) The sensory translation from rheological and IDDSI targets to consumer acceptance across diverse cultural food norms for dysphagia populations represents an underdeveloped bridge between food science and clinical nutrition.

## 7. Conclusions

This review provides a focused synthesis of heat-induced legume protein–starch gels by integrating three variables that have often been discussed separately: legume storage protein composition, starch structural properties and protein-to-starch ratio. Its main contribution is a three-axis compositional framework that links the legumin-to-vicilin ratio, starch amylose content and granule integrity, and phase-dominant formulation ratio to mixed-gel architecture and food functionality. Legumin-rich systems generally favour firmer and more cohesive gels, whereas vicilin-rich systems tend to require greater starch-side reinforcement; however, this protein axis must be interpreted together with extraction history, hydration state and processing conditions.

Starch provides a second structural lever through amylose content, granule integrity, swelling behaviour and retrogradation during cooling. Depending on botanical source and formulation ratio, starch can act as a filler, binder or secondary gel-forming phase. Together, these variables determine whether the final structure is protein-continuous, starch-continuous or bicontinuous, thereby influencing rheology, texture, water-holding capacity and starch digestibility.

By positioning plant-based meat analogues and dysphagia-oriented foods within the same compositional design space, this framework shows that these applications are not separate formulation problems, but contrasting endpoints of the same protein–starch structure–function continuum. Future work should prioritise underexplored chickpea and lentil systems, tribological evaluation beyond pea-based models, and mechanistic studies linking phase architecture to starch digestibility and storage stability.

## Figures and Tables

**Table 2:** Comparative properties of legume starches versus common cereal and tuber benchmarks.

Starch Source	Amylose (%)	Crystallinity Type	Peak Gelatinisation Temperature, *Tp* (°C)	Viscosity (RVA, Relative)	Retrogradation Tendency	References
*Pea (Pisum sativum)*	31–49	C	63.5–70.1	Moderate/high final viscosity	High	[63]
*Faba bean (Vicia faba)*	31–40	C	64.2–71.3	Moderate	High	[63]
*Chickpea (Cicer arietinum)*	30.4	C	65.3	Moderate/lowest PV among tested legumes	High (with amylose–lipid V-complexes)	[65]
*Lentil (Lens culinaris)*	32–39	C	65.2–70.3	Moderate	High	[63]
*Mung bean (Vigna radiata)*	30–45	C	67.4 or ~63–76	Very high; high breakdown	High but brittle/strong retrogradation	[16,66,67,68]
Wheat starch (cereal benchmark)	~17–25	A	~58–65	Moderate swelling and pasting viscosity	Low–moderate	[69,70,71]
Potato starch (tuber benchmark)	~20–30	B	~60–70	High swelling and paste viscosity	High	[69,70,71]

Abbreviations: *Tp*, peak gelatinisation temperature; RVA, Rapid Visco Analyser. A-, B-, and C-type crystallinity refer to starch chain packing patterns determined by X-ray diffraction.

## Data Availability

Data available on request.
